# High-Fat Diet-Induced Renal Proximal Tubular Inflammatory Injury: Emerging Risk Factor of Chronic Kidney Disease

**DOI:** 10.3389/fphys.2021.786599

**Published:** 2021-12-07

**Authors:** Shuxian Chen, Jinxia Chen, Shangmei Li, Fengbiao Guo, Aifen Li, Han Wu, Jiaxuan Chen, Quanren Pan, Shuzhen Liao, Hua-feng Liu, Qingjun Pan

**Affiliations:** Key Laboratory of Prevention and Management of Chronic Kidney Disease of Zhanjiang City, Affiliated Hospital of Guangdong Medical University, Zhanjiang, China

**Keywords:** high-fat diet, renal proximal tubules, inflammation, mechanisms, chronic kidney disease, dyslipidemia

## Abstract

Nowadays, with the improvements in living standards and changes in living habits, high-fat diet (HFD) has become much more common in the populations worldwide. Recent studies have shown that HFD could induce lipid accumulation, and structural and functional abnormalities, accompanied by the release of large amounts of pro-inflammatory cytokines, in proximal tubular epithelial cells (PTECs). These findings indicate that, as an emerging risk factor, PTEC injury-induced by HFD may be closely related to inflammation; however, the potential mechanisms underlying this phenomenon is still not well-known, but may involve the several inflammatory pathways, including oxidative stress-related signaling pathways, mitochondrial dysfunction, the myeloid differentiation factor 2/Toll like receptor 4 (MD2/TLR4) signaling pathway, the ERK1/2-kidney injury molecule 1 (KIM-1)-related pathway, and nuclear factor-κB (NF-κB) activation, etc., and the detailed molecular mechanisms underlying these pathways still need further investigated in the future. Based on lipid abnormalities-induced inflammation is closely related to the development and progression of chronic kidney disease (CKD), to summarize the potential mechanisms underlying HFD-induced renal proximal tubular inflammatory injury, may provide novel approaches for CKD treatment.

## Introduction

Chronic kidney disease (CKD) is a non-communicable disease with a high prevalence; it eventually presents as renal fibrosis, which is characterized by glomerulosclerosis, tubular atrophy, and tubular interstitial fibrosis (TIF; Hill et al., 2016; Lv and Zhang, 2019). The prevalence of CKD was about 9.1% in the world’s population and the global mortality was up to 1.2 million in 2017 (Bikbov et al., 2020).CKD can be induced by primary and secondary glomerulonephritis, renal tubular injury, and renal vascular lesions, such as diabetic nephropathy (DN), renal tubulointerstitial disease, and ischemic nephropathy (Lin et al., 2014; Moreno et al., 2018; Dong et al., 2019). CKD is caused by various conditions, including inflammation, oxidative stress, metabolic disorders, microvascular damage, and nephron loss (Moreno et al., 2018; Meng, 2019; Rapa et al., 2019; Ding et al., 2020; Juszczak et al., 2020; Ruiz-Ortega et al., 2020); however, the molecular mechanisms of CKD pathogenesis remain unclear. It has been reported that CKD induces multiple complications, including anemia, chronic kidney disease-mineral bone disease (CKD-MBD), and cardiovascular disease, and leads to reduced quality of life and even death (Webster et al., 2017). End-stage renal disease (ESRD), which requires renal replacement therapies such as kidney transplantation and dialysis to keep patients alive, is the final stage of CKD, and causes a heavy financial burden on the patient and the society in terms of public medical resources (Wang et al., 2016). As at now, CKD morbidity and mortality are still on the rise, making it an important public health concern. However, patients with CKD respond poorly to treatment. Therefore, for the elaboration of effective treatment strategies for CKD, it is important to clarify its pathogenesis.

High-fat diet (HFD) refers to the consumption of high fat content foods; many studies have shown that HFD is negatively associated with human health (Hohos and Skaznik-Wikiel, 2017; Rohr et al., 2020; Zimmerman et al., 2021) and that HFD is positively correlated with the occurrence of many diseases, including obesity, cardiovascular disease, and nonalcoholic fatty liver disease (NAFLD; Rabinovich-Nikitin et al., 2019; Gao et al., 2020; Gong et al., 2020; Zhang et al., 2020b). Several recent studies have reported that HFD could induce renal proximal tubular injury (Szeto et al., 2016; Yamamoto et al., 2017; Xu et al., 2019), and this may be associated with the activation of inflammatory pathways (Kennedy et al., 2013). These findings indicate that HFD may cause CKD through renal proximal tubular inflammatory injury. This review summarizes the potential role of HFD in CKD pathogenesis. We hope it would provide a scaffold for further studies on CKD pathogenesis and provide a potential theoretical basis and new direction for clinical CKD treatment.

## Role of Lipids in Renal Proximal Tubular Physiological Function

Renal proximal tubules are the main components of the renal tubules and play a pivotal role in reabsorption and excretion. The maintenance of lipid homeostasis is essential for renal proximal tubular physiological function (Gullans et al., 1984; Balaban and Mandel, 1988).

Lipids are the fundamental composition of cells. They present in the kidneys include triglycerides, phospholipids, cholesterol and its esters, and free fatty acids (FFAs; Hagve et al., 2001; Herman-Edelstein et al., 2014; Chen et al., 2019). In mammalian cells, FFAs are generated through the *de novo* synthetic pathway and released when triglycerides and phospholipids are hydrolyzed by cellular lipases (Schaffer, 2003). FFAs can also be imported into mammalian cells by both protein- and non-protein-mediated mechanisms (Schaffer, 2002). Most FFAs bind to albumin in plasma, and albumin-bound FFAs can be absorbed in the proximal tubules through receptor-mediated albumin endocytosis (Bobulescu, 2010). In addition, proximal tubular epithelial cells (PTECs) take up circulating FFAs dissociated from albumin through specific membrane proteins, such as fatty acid (FA) translocase (CD36) and FA-binding protein (Stremmel et al., 2001). These absorbed lipids are then oxidized and metabolized in PTEC mitochondria to produce ATP, thereby, maintaining energy balance in the tubules (Brunskill et al., 1997; Birn and Christensen, 2006; Kang et al., 2015; Yamamoto et al., 2021). Under physiological conditions, lipid metabolism in PTECs may protect renal tubules from damage caused by lipid overload. However, excess lipid accumulation may lead to renal tubular damage in the proximal tubules.

## Dyslipidemia and Renal Proximal Tubular Injury

### Dyslipidemia in CKD Patients

Dyslipidemia, which is caused by various factors, refers to the pathological accumulation of abnormal lipids and their metabolites in blood, tissues, and organs. It is characterized by the abnormal concentration of triglyceride (TG), cholesterol such as total cholesterol (TC), very-low-density lipoprotein cholesterol (VLDL-C), low-density lipoprotein cholesterol (LDL-C), intermediate-density lipoprotein cholesterol (IDL-C), and high-density lipoprotein cholesterol (HDL-C), or other lipids (Catapano et al., 2016; Kopin and Lowenstein, 2017). The dyslipidemia in CKD patients showed the elevated concentration of TG, VLDL, and IDL, the reduced concentration of HDL-C and the varied concentration of TC and LDL-C (Hager et al., 2017; Lamprea-Montealegre et al., 2018; Miljkovic et al., 2018; Bermudez-Lopez et al., 2019; Czaplińska et al., 2019). Many evidences suggested that lipids are nephrotoxic. The cholesterol levels are associated with the elevated mortality in the absence of inflammation and malnutrition in dialysis patients (Liu et al., 2004). The increased TC, non-HDL-C and the ratio of TC/HDL levels, and decreased HDL are significantly related to renal dysfunction in men with an initial creatinine <1.5 mg/dl (Schaeffner et al., 2003). Further, a Chronic Renal Insufficiency Cohort (CRIC) study showed that LDL-C and TC concentration are inversely associated with kidney disease outcomes in patients with low proteinuria levels (Rahman et al., 2014). The elevated levels of LDL receptor (LDLR), oxidized LDL (ox-LDL), acetylated LDL (acLDL), and CD-36 are significantly related to the progression of DN and deterioration of eGFR (Kiss et al., 2013; Herman-Edelstein et al., 2014). Previous studies have shown that patients with nephrotic syndrome are usually comorbid with lipid metabolic disorders characterized by elevated serum cholesterol, TG, and phospholipid levels, and decreased serum HDL levels (Joven et al., 1990; Vaziri, 2016). CKD patients with significant proteinuria may also suffer from dyslipidemia and have increased serum creatinine (Scr) levels (Agrawal et al., 2018). Renal biopsy specimens from patients with DN showed lipid deposition in renal inherent cells, especially in PTECs, as well as renal structural and functional abnormalities (Bobulescu, 2010; Kiss et al., 2013; Herman-Edelstein et al., 2014). These results confirmed that lipid accumulation in kidney is associated with abnormal renal function. Study reported that PTECs appear to be more prone to lipid accumulation than other renal inherent cells, probably due to their higher energy expenditure rate (Gai et al., 2019). Dyslipidemia in patients with CKD is associated with a deterioration of renal function (Herman-Edelstein et al., 2014). The “lipid nephrotoxicity” hypothesis proposed by Moorhead et al. (1982) stated that dyslipidemia could affect CKD progression; this hypothesis has aided our understanding of CKD progression. Recently, lipid nephrotoxicity became a significant research hotspot.

### HFD-Induced Renal Proximal Tubular Injury *in vivo*

Lipid abnormalities are detrimental to the kidneys. A study showed that HFD alone could not induce kidney lesions (Yang et al., 2017); however, a large amount of data indicated that HFD increases lipid accumulation in proximal tubules and lipid accumulation (especially saturated fatty acid, SFA)-induced alternations in the function and structure of PTECs (Arici et al., 2003; de Vries et al., 2014; Declèves et al., 2014; Khan et al., 2014; Li et al., 2016; Yamamoto et al., 2017). These findings are summarized in Table 1.

**Table 1:** Relevant information on high-fat diet (HFD)-induced renal proximal tubular impairment.

	Feature	References
Functional impairment	(1) Elevated Scr and BUN levels; (2) Creased inflammatory cytokine expression; (3) Boosted levels of the kidney injury; (4) Biomarkers: NGAL, renin mRNA, and KIM-1; (5) Oxidative stress, mitochondrial dysfunction, and impairment of lysosomal acidification.	Declèves et al., 2014; Fang et al., 2015, 2017; Yamamoto et al., 2017; Cheng et al., 2019; Xu et al., 2019; Hewage et al., 2020; Sun et al., 2020
Structural lesions	(1) Lipid accumulation and vacuolar degeneration: the cytoplasm is filled with vacuoles containing; (2) Multilamellar, onion skin-like structures; (3) Brush border impairment; (4) Epithelial cell detachment, tubular dilatation, and tubulointerstitial extracellular matrix accumulation.	Hao et al., 2012; Declèves et al., 2014; Kuwahara et al., 2016; Szeto et al., 2016; Yamamoto et al., 2017; Takagi et al., 2018; Sun et al., 2020

Scr, serum creatinine; BUN, blood urea nitrogen; NGAL, neutrophil gelatinase-associated lipocalin; mRNA, messenger RNA; and KIM-1, kidney injury molecule 1.

Increasing evidence indicated that HFD induces an increase in cholesteryl ester and phospholipid levels in proximal tubules, and a significant elevation in Scr and blood urea nitrogen (BUN) levels, pro-inflammatory cytokines production, and kidney injury biomarker production, including the neutrophil gelatinase-associated lipocalin (NGAL), renin messenger RNA (mRNA), and kidney injury molecule 1 (KIM-1; Declèves et al., 2014; Fang et al., 2015, 2017; Cheng et al., 2019; Luo et al., 2019; Xu et al., 2019; Hewage et al., 2020). Oxidative stress, mitochondrial dysfunction, and impairment of lysosomal acidification were observed in PTECs following HFD treatment (Yamamoto et al., 2017; Sun et al., 2020). These results suggested that HFD may impair renal function.

High-fat diet can also induce the destruction of the renal proximal tubular structure (Luo et al., 2019). Studies have reported an increase in vacuole number in mouse renal PTECs, accompanied by impaired brush border and epithelial cell shedding, following HFD treatment (Declèves et al., 2014; Li et al., 2016; Sun et al., 2020). Following HFD treatment, mouse renal PTEC vacuoles were found to contain multilamellar, onion skin-like structures (Declèves et al., 2014; Kuwahara et al., 2016; Yamamoto et al., 2017). In addition, several studies have reported the filling of PTECs with lipid droplets following HFD treatment (Hao et al., 2012; Szeto et al., 2016; Muller et al., 2019). These results indicate that HFD induces lipid accumulation and vacuolar degeneration in PTECs. HFD can also induce epithelial cell detachment, tubular dilatation, and tubulointerstitial extracellular matrix accumulation (Hao et al., 2012; Takagi et al., 2018). HFD-induced renal proximal tubular injury occurs mainly in the S1 and S2 renal tubules, and this may be related to the fact that these tubules are rich in mitochondria, lysosomes, and other organelles (Declèves et al., 2014).

In addition, HFD was found to be associated with significant disorganization of the cytoplasm and mitochondrial damage (Szeto et al., 2016; Takagi et al., 2018). These mitochondria appeared small and round, were in constant fission, and had no cristae membranes as opposed to those in cells of mice fed with normal diet (Szeto et al., 2016; Sun et al., 2020).

Interestingly, the adverse impact of maternal exposure to HFD on the kidneys of offspring has been confirmed by some studies (Nguyen et al., 2019; Shamseldeen et al., 2019; Larkin et al., 2021). Chowdhury et al. (2016) reported that in rats, paternal exposure to HFD could lead to renal lipid accumulation, loss of brush border, and increased cell sloughing in adult offspring; however, these effects were mild. Further studies are needed to determine whether HFD induces alterations in the kidneys of offspring.

### Palmitic Acid-Induced Renal Tubular Epithelial Cell Lesions *in vitro*

Many studies have shown that treatment with SFAs, especially palmitic acid (PA), can damage cultured human PTECs (HK-2 cells; Xie et al., 2017; Yamamoto et al., 2017; Qiu et al., 2018). Endoplasmic reticulum (ER) stress, increased lipid deposition, cell apoptosis, and an elevated inflammatory response have been observed in PA-treated HK-2 cells (Wu et al., 2019; Mai et al., 2020). Furthermore, PA-treated rat PTECs (NRK-52E cells) showed a significant increase in the levels of cleaved caspase-3, which is significantly associated with apoptosis (Cobbs et al., 2019). In addition, a significant increase and decrease in mitochondrial reactive oxygen species (ROS) and mitochondrial oxidative phosphorylation gene mRNA levels, respectively, were observed in PA-treated PTECs, confirming that HFD could induce mitochondrial injury (Yamamoto et al., 2017). Moreover, cell survival was found to be decreased in HFD-treated renal PTECs (Yamamoto et al., 2017). These findings provided evidence of the harmfulness of HFD in renal PTECs; however, the molecular mechanisms underlying this is unclear.

## HFD-Induced Renal Tubular Inflammatory Injury and Its Potential Underlying Mechanisms

**Figure 1:**
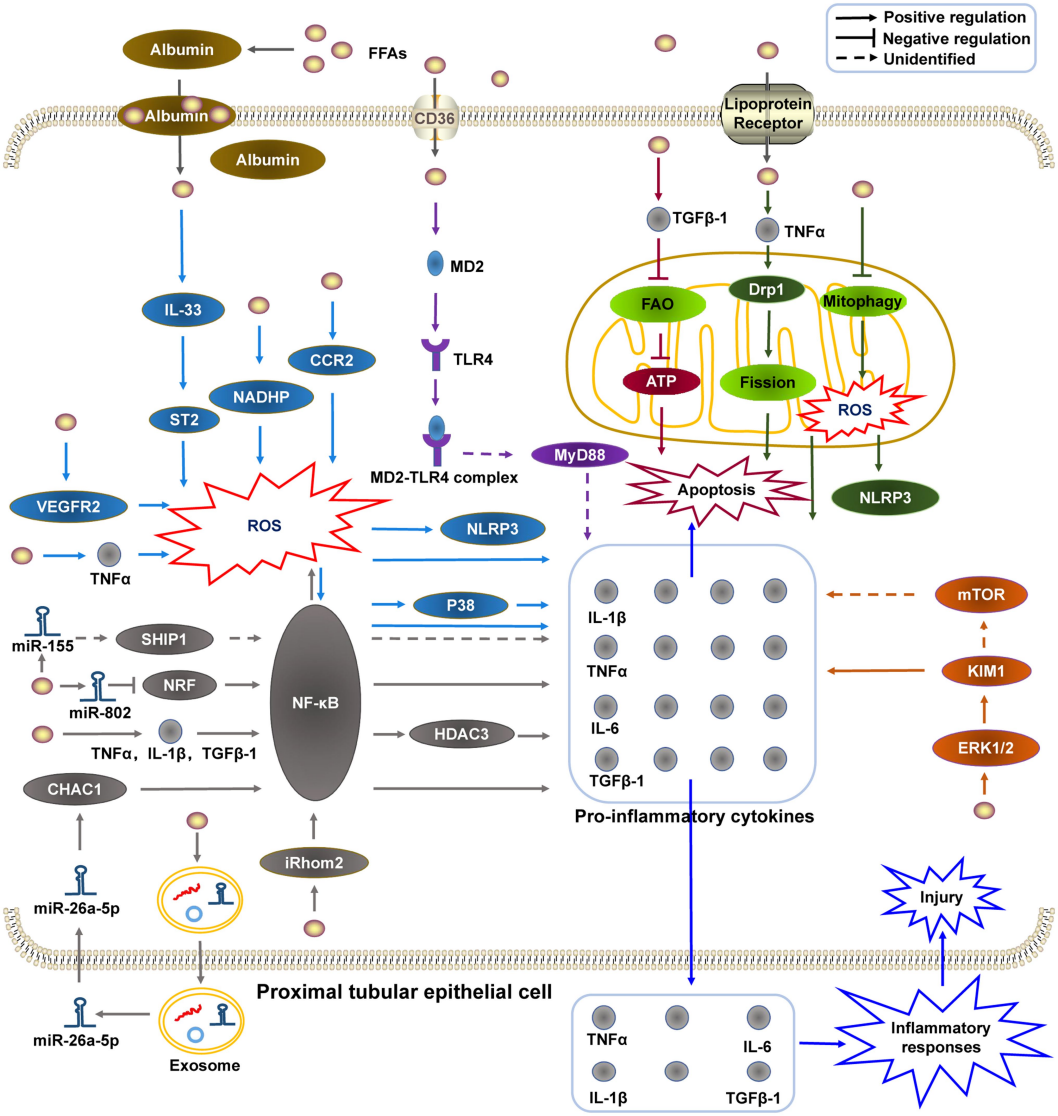
The potential mechanisms of HFD-induced renal proximal tubular epithelial cell inflammatory injury. (1) HFD promotes the generation of pro-inflammatory molecules by stimulating the MD2-TLR4 complex to recruit MyD88; (2) HFD-induced mitochondrial dysfunction is related to mitochondrial FAO impairment, mitophagy deficiency, and dynamic change; (3) KIM-1 may be upregulated by HFD-activated ERK1/2, and promotes the release of pro-inflammatory molecules directly or indirectly through the mTOR-related pathway; (4) HFD induces NF-κB activation *via* different pathways (e.g., miRNAs), and subsequently upregulates the expression of pro-inflammatory cytokines; and (5) Oxidative stress participate in HFD-induced PTEC inflammatory injury *via* different pathways. HFD, high-fat diet; MD2-TLR4, myeloid differentiation molecule 2–toll like receptor 4; MyD88, myeloid differentiation primary response protein 88; FAO, fatty acid oxidation; KIM-1, kidney injury molecule 1; ERK1/2, extracellular signal-regulated kinase 1/2; mTOR, mammalian target of rapamycin; NF-κB, nuclear factor-κB; and miRNAs, micro RNAs.

Chronic inflammation and lipid abnormalities are important synergistic factors that induce pathological changes in the kidneys (Wang et al., 2018). Aside from playing a vital role in reabsorption, renal PTECs also function as inflammatory cells and release various pro-inflammatory cytokines in response to injury (Liu et al., 2018). Many recent studies have shown that HFD triggers renal PTEC injury, accompanied by monocyte infiltration and the release of inflammatory signaling molecules, such as interleukin (IL-1β, IL-6), tumor necrosis factor alpha (TNFα), transforming growth factor β1 (TGF-β1), chemokines, such as monocyte chemotactic protein 1 (MCP-1), and IL-8 (Kim et al., 2013; Szeto et al., 2016; Yamamoto et al., 2017; Yang et al., 2017; Wang et al., 2017b; Hewage et al., 2020). Inflammatory cytokines protect the renal tubules from damage during the early stages of injury (Meng et al., 2014), while chronic inflammatory infiltration may damage the structure and function of renal tubules at an advanced stage, subsequently causing renal tubular fibrosis (Meng, 2019). Furthermore, HFD-induced excessive SFA accumulation (especially PA, which accounts for 80–90% of SFAs) in renal PTECs can stimulate the production of pro-inflammatory cytokines and adhesion molecules, and induce the activation of the nuclear factor kappa B (NF-κB) signaling pathway, thus causing renal tubular dysfunction and inflammatory damage (Huang et al., 2012; Fang et al., 2017). Long-term pro-inflammatory cytokines infiltration can induce TIF progression. The specific signaling pathways involved in inflammation and tubular lipid toxicity, which may be related to the inflammatory pathways below (Figure 1), need to be further elucidated. Various studies indicated that several signaling pathways including oxidative stress-related signaling pathways, mitochondrial dysfunction-related signaling pathways, the myeloid differentiation molecule 2 (MD2)-toll-like receptor 4 (TLR4) signaling pathways, the ERK1/2-KIM-1-related pathways, and NF-κB activation were involved in HFD-induced renal inflammatory injury.

### Oxidative Stress-Related Signaling Pathways

As known, oxidative stress is detrimental to cells due to the ROS and reactive nitrogen species (RNS) overproduction. To date, many studies have suggested that HFD-induced oxidative stress is detrimental to kidney (Arany et al., 2016; Mei et al., 2020; Sun et al., 2020).

High-fat diet significantly promote the production of ROS and inflammatory response such as NLRP3 activation and the release of many inflammatory cytokines in kidney (Morris et al., 2009; Yamamoto et al., 2017; Weihong et al., 2019; Zheng et al., 2019b; Sun et al., 2020). The production of ROS is the critical event of oxidative stress (Muthulakshmi and Saravanan, 2013), which plays a key role in the pathogenesis of CKD (Daenen et al., 2019; Neelofar et al., 2019). HFD-induced oxidative stress in renal tubules is characterized by the increased production of ROS and NADPH oxidase such as oxidative stress marker Gp91, and thus promote the cytochrome C release from mitochondria to cytoplasm (Sun et al., 2020).

Moreover, recent studies confirmed that ROS may lead to the inflammatory injury of renal tubules in HFD (Song et al., 2018; Rehman et al., 2019; Zheng et al., 2019b; Eleazu et al., 2020). A study showed that the inhibition of ROS production may block the secretion of pro-inflammatory cytokines *via* inhibiting NF-κB signal pathway (Rehman et al., 2019). Another study showed that HFD-induced renal tubular inflammatory injury and oxidative stress is associated with iRhom2/NF-κB and Nrf-2/HO-1 signaling pathways (Chenxu et al., 2021). Furthermore, HFD-induced renal injury is related to the expression of NADHP oxidase, which may active ROS-mediated NLRP3 inflammasome and NF-κB/p38 signaling pathway (Song et al., 2018). In addition, HFD may cause oxidative stress and renal proximal tubular injury through IL-33/ST2 signal pathway (Elsherbiny et al., 2020). The upregulated expression of HFD-induced vascular endothelial growth factor receptor-2 (VEGFR2) in the kidney may induces NLRP3-dependent inflammatory responses *via* increasing ROS production (Zheng et al., 2019b). CCR2-knockout may improve renal injury through inhibiting oxidative stress and ER stress in HFD (Lee et al., 2019). TNF-α deficiency also can downregulate the oxidative stress and inflammation of proximal tubular cells in HFD (Wang et al., 2017a). Collectively, these results indicated that HFD-induced oxidative stress may lead to the renal injury *via* different signal pathways.

In summary, the production of ROS is involved in the pathogenesis of renal inflammatory injury-induced by HFD, and ROS target therapy can significantly inhibit dyslipidemia and improve renal inflammatory injury.

### Mitochondrial Dysfunction

As known, mitochondria mainly provide ATP and energy to maintain cellular homeostasis and regulate the communication between cells and tissues. Recent studies showed that mitochondria play an important role in HFD-induced renal inflammatory injury (Sun et al., 2020; Han et al., 2021; Lubojemska et al., 2021). Mitochondrial dysfunction-induced by HFD is related to mitochondrial fatty acid oxidative (FAO) impairment, dynamic change (e.g., mitochondrial fission and fusion) and mitophagy deficiency.

Insufficient mitochondrial FAO in renal resident cells is thought to be responsible for HFD-induced kidney injury (Xu et al., 2014; Kang et al., 2015; Lv et al., 2019). Previous studies have found that HFD could lead to mitochondrial FAO dysfunction and promote the expression of transforming growth factor beta 1 (TGFβ1; Wang et al., 2018; Lv et al., 2019). TGFβ1 was found to be an important upstream regulator of FA metabolism in PTECs (Kang et al., 2015). These findings show that FAO impairment is involved in HFD-induced renal PTEC injury.

Long-term lipid overload may lead to phospholipid accumulation and inadequate acidification in lysosomal system of PTECs, stagnates autophagic flux, subsequently resulting in the deficiency of renal tubular autophagy (Yamamoto et al., 2017). The production of ROS in mitochondria is increased significantly and the ratio of mitochondrial DNA/nuclear DNA is decreased significantly in PTECS with autophagy deficiency, and thus induce inflammatory response (Yamamoto et al., 2017). For the molecular mechanisms involved in this progress, Unc-51-like autophagy activating kinase 1 (ULK1) has been confirmed to be inactivated in HFD-induced renal injury (Declèves et al., 2011, 2014; Sohn et al., 2017; Luo et al., 2019). More important, the recovery of mitophagy deficiency can attenuate HFD-induced renal tubular inflammatory injury (Han et al., 2021). In addition, mitochondrial dynamic changes were also reported in HFD (Chen et al., 2018; Sun et al., 2020).

Collectively, these findings show that FAO impairment, mitophagy deficiency and mitochondrial dynamic changes play pathogenic roles in HFD-induced renal inflammatory injury.

### The MD2-TLR4 Signaling Pathway

Toll-like receptor 4 is a pattern recognition receptor and an important innate immune regulator that stimulates innate immune responses by binding to a series of infectious agent-associated ligands, such as the MD2, which is an important pathogenic factor that contributes to inflammatory injury, and is required for TLR4 activation (Takeuchi and Akira, 2010; Huang et al., 2012; Rocha et al., 2016). MD2 initially binds to the cell surface ligand and then induces the activation of the MD2-TLR4 immune signaling complex (Wang et al., 2017b). Data indicated that the MD2-TLR4 complex is a key pathogenic agent in the progression of obesity-related nephropathy (Xu et al., 2019). Several recent studies have shown that HFD induces MD2 expression and promotes the formation of the MD2-TLR4 complex in renal tissues, thereby causing inflammation and injury in PTECs (Fang et al., 2017; Xu et al., 2019; Zhang et al., 2020a). MD2 inhibition or knockout was found to prevent SFA-induced renal PTEC inflammation and injury. However, the specific target of the MD2-TLR4 signaling pathway-mediated HFD-induced renal tubular inflammatory injury still needs to be determined. Moreover, many studies suggested that FFAs induces NF-κB to promote the release of pro-inflammatory cytokines (e.g., TNFα, IL-6, and IL1β) by stimulating the MD2-TLR4 complex to recruit myeloid differentiation primary response protein 88 (MyD88; Rocha et al., 2016; Wang et al., 2017b; Song et al., 2021). However, whether this pathway participates in lipid nephrotoxicity remains to be further verified.

Oxidized low-density lipoprotein (ox-LDL), which is metabolized to 9-hydroxyoctadecenoic acid and 13-octadecenoic acid, is an important biomarker of oxidative lipid damage, and activates the peroxisome proliferator-activated receptor γ (PPARγ), a transcription factor involved in adipogenesis (Gai et al., 2019). This implies that ox-LDL may mediate tissue oxidative damage by activating transcription factors associated with lipid production. HFD significantly promotes ox-LDL generation. Xu et al. (2019) initially reported that ox-LDL-induced MD2-mediated TLR4 activation induces PTEC lesions in rats, and that the inhibition of MD2 blockade effectively prevents ox-LDL- and HFD-induced lesions. This is a new discovery as concerns the potential factors involved in HFD-induced renal injury. In addition, PA induces the generation of pro-inflammatory cytokines by stimulating the MD2-TLR4 complex to recruit MyD88, which is primarily associated with FFA-induced inflammatory responses (Rocha et al., 2016; Wang et al., 2017b). Data has shown that three PA molecules bind to the MD2 capsule with a relatively high affinity to form a stable complex (Wang et al., 2017b). MD2 also binds to unsaturated fatty acids, but can only induce a pro-inflammatory response when it binds to SFAs (Wang et al., 2017b).

Therefore, it is necessary to understand how MD2 regulates ox-LDL-induced proximal tubule injury as this could provide a new reference for CKD treatment. However, there are few relevant studies on the subject, and this study was only conducted on cells, and findings thereof need to be further verified through animal experiments.

### The ERK1/2-KIM-1-Related Pathway

Kidney injury molecule 1, a type 1 transmembrane protein, is a sensitive proximal tubular injury marker (Han et al., 2002; Humphreys et al., 2013), which is expressed during PTEC impairment (van Timmeren et al., 2006; Ichimura et al., 2008; Gauer et al., 2016). HFD stimulates KIM-1 expression in PTECs and induces an increase in KIM-1-regulated proximal tubular inflammation and cell damage (Zhao et al., 2019), and this is a new discovery as far as lipid nephrotoxicity is concerned.

Studies have shown that KIM-1 protects the kidneys from further injury by regulating PTEC phagocytosis and through its anti-inflammatory effects during the early stages of kidney injury (Ichimura et al., 2008; Yang et al., 2015). However, KIM-1 overexpression may induce inflammation and interstitial fibrosis in renal tubules (van Timmeren et al., 2007; Humphreys et al., 2013; Lin et al., 2014). KIM-1 can be detected in large quantities in the plasma and urine samples of diabetic patients, and this may be correlated with the occurrence and development of early DN (Nowak et al., 2016). Researchers found that SFA accumulation can stimulate inflammation and KIM-1 overexpression in PTECs *in vitro* (Zhao et al., 2019). Therefore, KIM-1 may be significantly associated with lipid toxicity-induced inflammation and tubular injury. They further confirmed that KIM-1 was upregulated through extracellular signal-regulated kinase 1/2 (ERK1/2) activation, and promoted the expression of injury-related molecules such as osteopontin (OPN) and CD44, and cysteine aspartic protease 3; KIM-1 inhibition was found to alleviate PA-induced proximal tubular injury (Zhao et al., 2019). This study provided a new direction for the study of lipid nephrotoxicity. A study carried out in zebrafish models showed that sustained KIM-1 expression in proximal tubules induces the activation of the mammalian target of rapamycin pathway (mTOR), which mediates the loss of tubular brush border, subsequently triggering inflammation and the occurrence and progression of chronic kidney disease (Yin et al., 2016). Therefore, renal injury-induced by HFD-mediated KIM-1 upregulation may also be involved in the activation of the mTOR pathway.

Although many studies have confirmed that KIM-1 is related to kidney injury, the molecular mechanisms underlying the KIM-1-specific signaling pathway-mediated HFD-induced proximal tubular inflammatory injury, which leads to CKD, is yet to be elucidated.

### NF-κB Activation

Nuclear factor kappa B, which is composed of a series of transcription factors, is highly expressed in almost all cells, and can be activated by pro-inflammatory cytokines, such as TNF-α and IL-1 (Bonizzi and Karin, 2004; Pflug and Sitcheran, 2020). Its downstream target genes include IL-1β, IL-6, and TNFα, which play a pivotal role in regulating the inflammatory response (Oeckinghaus and Ghosh, 2009). In general, NF-κB activity is inhibited by the inhibitor of NF-κB proteins (IκBs) in the cytoplasm. NF-κB disintegrates and regulates nuclear transfer when exposed to external stimuli, thereby acting as a transcription factor and promoting the expression of various pro-inflammatory cytokines (Oeckinghaus and Ghosh, 2009).

It has been reported that NF-κB expression levels significantly increase in renal tubular cells following HFD treatment (Li et al., 2020); this subsequently induces an upregulation of the expression levels of pro-inflammatory cytokines such as IL-6 and TNFα (Hewage et al., 2020). Moreover, many studies have shown that in mice, NF-κB inhibition protects the kidneys from HFD-induced injury (Fang et al., 2015; Eleazu et al., 2020). However, it is still unclear which signaling pathways regulate the inflammatory response in HFD-induced renal tubular inflammation. A recent study showed that HFD promoted the releases of circulating pro-inflammatory cytokines (e.g., TNF-α, IL-1β, IL-6, and MCP-1), and enhanced the PTEC inflammation by activating NF-κB/histone deacetylase 3 (NF-κB/HDAC3) signaling pathway (Li et al., 2021).

Recently, miRNAs were reported that may significantly associated with the HFD-induced chronic renal inflammatory response (Sun et al., 2019; Zheng et al., 2019a). miR-155 was reported to participate in HFD-induced renal inflammatory and injury *via* the regulation of SHIP1/NF-κB signaling pathway (Zheng et al., 2019a). MiR-802 can regulate the renal inflammatory injury in HFD mice by directly suppressing the NF-κB-repressing factor (NRF; Sun et al., 2019). Furthermore, HFD-induced renal tubular inflammatory injury can be attenuated by inhibiting the secretion of exosomes from renal PTECs by targeting the miR-26a-5p/CHAC1/NF-κB pathway (Li et al., 2020).

At present, the role of miRNAs in NF-κB activation in HFD-induced renal tubular inflammatory still need further study. However, we believe that with the rapid advancements in technological platforms such as cell sequencing technology, great progress will be made in this field; this would help provide important data and information to improve our understanding of HFD-induced CKD pathogenesis and allow for the development of an effective intervention strategy for the disease.

## Future Perspectives

The prevalence of HFD is increasing rapidly worldwide, and the injury of renal tubules induced by HFD strongly associated with CKD. It has been well-known that inflammation plays a crucial role in the injury of renal tubule-induced by HFD; however, the specific molecular mechanisms involved in this progress have not yet been well identified to date, although they may include several signaling pathways (e.g., oxidative stress signaling pathways, NF-κB activation). Therefore, potential therapies target these pathways may hold promise for the treatment of HFD-induced CKD. Here, we summarize the novel insights into the mechanisms of HFD-induced renal tubular inflammatory injury in HFD-induced CKD, and promising therapeutic strategies, which may help improve the understanding of the pathogenesis of CKD and provide novel therapies for CKD.

## Author Contributions

SC, JC, SL, and QiP wrote the manuscript and designed the figures. SC, FG, AL, HW, JC, QuP, SL, and H-fL revised the manuscript. All authors contributed to the article and approved the submitted version.

## Funding

This study was supported by National Natural Science Foundation of China (no. 82070757), the Project of “Dengfeng Plan” and Department of established positions for the Zhujiang Scholar from Guangdong Medical University, and Guangdong Basic and Applied Basic Research Foundation (no. 2019A1515012203), and the Zhanjiang City Program for Tackling Key Problems in Science and Technology (no. 2019B01179).

## Conflict of Interest

The authors declare that the research was conducted in the absence of any commercial or financial relationships that could be construed as a potential conflict of interest.

## Publisher’s Note

All claims expressed in this article are solely those of the authors and do not necessarily represent those of their affiliated organizations, or those of the publisher, the editors and the reviewers. Any product that may be evaluated in this article, or claim that may be made by its manufacturer, is not guaranteed or endorsed by the publisher.
